# HIV-1 tolerates changes in A-count in a small segment of the pol gene

**DOI:** 10.1186/s12977-017-0367-0

**Published:** 2017-09-05

**Authors:** Bep Klaver, Yme van der Velden, Formijn van Hemert, Antoinette C. van der Kuyl, Ben Berkhout

**Affiliations:** Laboratory of Experimental Virology, Department of Medical Microbiology, Academic Medical Center, University of Amsterdam, K3-110, Meibergdreef 15, 1105 AZ Amsterdam, The Netherlands

**Keywords:** HIV-1 evolution, RNA genome, Nucleotide composition, Evolution, A-rich, Subtypes, Mutagenesis, Phylogeny, Silent codon changes

## Abstract

**Background:**

The HIV-1 RNA genome has a biased nucleotide composition with a surplus of As. Several hypotheses have been put forward to explain this striking phenomenon, but the A-count of the HIV-1 genome has thus far not been systematically manipulated. The reason for this reservation is the likelihood that known and unknown sequence motifs will be affected by such a massive mutational approach, thus resulting in replication-impaired virus mutants. We present the first attempt to increase and decrease the A-count in a relatively small polymerase (pol) gene segment of HIV-1 RNA.

**Results:**

To minimize the mutational impact, a new mutational approach was developed that is inspired by natural sequence variation as present in HIV-1 isolates. This phylogeny-instructed mutagenesis allowed us to create replication-competent HIV-1 mutants with a significantly increased or decreased local A-count. The local A-count of the wild-type (wt) virus (40.2%) was further increased to 46.9% or reduced to 31.7 and 26.3%. These HIV-1 variants replicate efficiently in vitro, despite the fact that the pol changes cause a quite profound move in HIV–SIV sequence space.

**Conclusions:**

Extrapolating these results to the complete 9 kb RNA genome, we may cautiously suggest that the A-rich signature does not have to be maintained. This survey also provided clues that silent codon changes, in particular from G-to-A, determine the subtype-specific sequence signatures.

## Background

It has been noticed since long that the RNA genome of the human immunodeficiency virus type 1 (HIV-1) has a significantly biased nucleotide composition [1]. The A-numbers reach 35.1% and the C-count is particularly low at 18.1% [2]. This A-pressure is rather strong and even influences the amino acid composition of the HIV-1 proteins with a preference for residues that are encoded by A-rich codons [3]. Two evolutionary scenarios have been proposed to explain this phenomenon. First, the biased nucleotide composition may be imposed over evolutionary times by a distinct mutational pattern of the viral polymerase. HIV-1 Reverse Transcriptase copies the RNA genome and indeed, at least under some conditions, introduces C-to-U mutations that result in G-to-A changes in the RNA genome [4, 5]. Alternatively, the cellular Apobec enzymes may have edited the HIV-1 RNA genome by G-to-A hypermutagenesis [6–8]. A second scenario entertains a functional role for the A-rich RNA genome. For instance, we previously suggested that this A-bias may create a distinctive signature that is recognized by the viral Gag protein during selective packaging of the viral RNA genome amongst a multitude of cellular transcripts [9]. There is some recent support for the latter scenario. The HIV-1 nucleocapsid (NC) domain of the Gag protein was reported to undergo a switch in RNA-binding mode during virion assembly: from recognizing GU-rich sequences as NC monomer to packaging of A-rich sequences by Gag multimers as part of the assembling virion [10, 11]. It was also proposed that the particular nucleotide composition of the invading HIV-1 RNA is critical for reverse transcription [12], that two A-rich domains within the Gag open reading frame direct translation events [13] and that it is detected by the innate immune system in human cells [14, 15]. Analysis of the A-richness is further complicated by other nucleotide pressures, e.g. strong suppression of the CG dinucleotide [16, 17], codon usage bias that is possibly coupled to restriction by the cellular Schlafen 11 protein [18] and a certain bias for codon-pairs [19].

Thus, it seems important to probe the replication capacity and molecular properties of modified HIV-1 variants with an altered A-count of the RNA genome. However, it is quite risky to massively increase or decrease the number of A nucleotides in the HIV-1 RNA genome because of the likelihood that multiple important sequence elements will be affected. For instance, packaging signals in the viral RNA are known to extend into the Gag open reading frame [20] and the HIV-1 genome is riddled with regulatory splice signals [21–23]. Here, we present a first conservative attempt to create such A-up/down HIV-1 variants. Several precautions were made to increase the chance of generating replication-competent virus variants. First, only silent codon changes were allowed in order not to change the encoded viral proteome. Second, only a small genome segment of 498 nucleotides was chosen for this round of A up/down mutagenesis, which represents some 5.5% of the complete genomic RNA. Third, we have chosen a relatively “relaxed” part of the HIV-1 genome for mutagenesis, e.g. a domain that does not encode overlapping open reading frames or important regulatory RNA signals, in order to minimize the chances of inducing replication defects. Fourth and likely most important, we implemented a new approach to select functionally neutral base substitutions, a method that we will call phylogeny-instructed mutagenesis (PIM). The resulting viruses were tested in single cycle assays that score the early phase (infectivity) and late phase (virus production) of the HIV-1 replication cycle and virus spread was measured in a transformed T cell line and primary T cells.

## Methods

### Synthetic HIV-1 DNA

We used numbering according to the HIV-1 LAI DNA plasmid map with the +1 being the first nucleotide of the LTR promoter [24, 25]. Restriction enzyme sites are numbered according to the first nucleotide position. Synthetic HIV-1 gene fragments encompassing the Bcl1-Bst1107I fragment (2512–3015) were synthesized by GeneArt Strings™ DNA Fragments. We cloned these fragments, digested them with Bcl1 and Bst1107I into the similarly digested pLAI plasmid that was isolated from the dam^−^ E. coli strain 3902. The complete inserts were verified by sequence analysis.

### Virus production

Human embryonic kidney (HEK) 293T cells were cultured in DMEM (Invitrogen) supplemented with 10% fetal bovine serum (FBS), non-essential amino acids (0,1 mM, Invitrogen), 100 U/ml penicillin and 100 U/ml streptomycin at 37 °C and 5% CO_2_. Cells were grown in 9.5 cm^2^ wells and transfected by the calcium phosphate method with 5 μg wt or mutant pLAI-based construct, as previously described [26]. Two days post transfection, the supernatant was harvested and the supernatant CA-p24 level was determined by ELISA [27, 28].

### RT activity assay

Virus stocks were produced in transfected 293T cells. The Reverse Transcriptase (RT) activity present in virion particles in the culture medium was determined with a real-time PCR-based RT assay that used avian myeloblastosis virus (AMV) RT as standard [29].

### Single cycle infection TZM-bl assay

These cells express the HIV-1 receptors CD4, CCR5 and CXCR4 and contain the luciferase gene under control of the HIV-LTR promoter. TZM-bl cells are routinely used to measure HIV-1 infectivity. Cells were cultured and maintained in DMEM supplemented with 10% FBS, non-essential amino acids (Invitrogen), 100 U/ml penicillin and 100 µg/ml streptomycin at 37 °C and 5% CO_2_. Cells were grown to 60% confluency in 0.95 cm^2^ wells and infected with wt or mutant 293T-produced virus (the equivalent of 0, 0.5, 5 or 50 ng CA-p24). Cells were washed with phosphate-buffered saline (PBS) after 2 days and lysed in passive lysis buffer (Promega). The firefly luciferase was measured in cell lysates with the luciferase assay kit (Promega).

### HIV-1 infections

The SupT1 T cell line (10^5^ cells) was infected with 293T-produced virus (equivalent of 2.5 ng CA-p24). To perform infections on CD4^+^ T lymphocytes, we first isolated peripheral blood mononuclear cells (PBMCs) from buffy coats (Sanquin) of healthy volunteers using Ficoll–Hypaque density centrifugation [30]. Cells of three donors (homozygous wild-type CCR5) were pooled and cultured in RPMI containing 10% FCS, 100 U/ml penicillin, 100 µg/ml streptomycin, 100U/ml recombinant IL-2 and the first 3 days also with 2 µg/ml phytohemagglutinin (PHA) to activate the T lymphocytes. At day 5 the CD4^+^ T lymphocytes were isolated by depleting the CD8^+^ population using CD8 Dynabeads (Invitrogen) according to manufacturer’s protocol. The CD4 T lymphocytes (4 × 10^5^) were infected at 37 °C with equal amounts of 293T-produced virus (equivalent of 5 ng CA-p24). Cells were maintained with 100 units/ml recombinant interleukin-2 following infection.

### Virus evolution

The protocol for virus evolution by prolonged cell-free passage of virus onto fresh, uninfected SupT1 cells was described previously [27]. SupT1 T cells were cultured in advanced RPMI 1640 medium supplemented with 1% (v/v) FBS, 100 U/ml penicillin, 100 U/ml streptomycin at 37 °C and 5% CO_2_. Cells (5 × 10^6^) were transfected with 1 μg DNA of the pLAI-based variants by means of electroporation (250 V, 975 μF) using a BioRad Gene Pulser II as previously described [31]. Cells were split 1–5 twice a week. The CA-p24 level in the culture medium was monitored by ELISA. Isolation of total cellular DNA from HIV-infected cells was performed by Tween20/Proteinase K treatment [32]. The pol region was PCR-amplified with primers gag2 (with codons marked CT.CTA.TTA.GAT.ACA.GGA.GCA.GAT.G) and ET43 (CTT.CTG.TAT.GTC.ATT.GAC.AGT). The PCR products were “directly” sequenced by BigDye terminator sequencing (Applied Biosystems, Foster City, CA), thus yielding the “population” sequence of the viral quasispecies.

## Results

### Design of the Low, Min and Max mutants

We decided to redesign a 498-nt HIV-1 pol gene fragment that encodes part of the RT enzyme by means of PIM. We choose the fragment encoded between the restriction sites Bcl1 (2512) and Bst1107I (3015) in the pLAI molecular clone to facilitate easy cloning. PIM used the sequence of 675 HIV-1 subtype B isolates present in the Los Alamos sequence compendium [33]. All sequences were aligned with the prototype LAI isolate and we scored the number of A, C, U and G nucleotides per position. Insertions and deletions, usually a multiple of 3-nt codons, were ignored. To restrict the mutagenesis to silent codon changes, we focused on 3rd codon positions. We selected natural sequence variation that differs from the LAI sequence for introduction in the LAI genome, but with the following thresholds. The Max variant incorporated all possible changes from a regular LAI residue into A when observed in at least 5 HIV-1 isolates (0.7%). This led to the introduction of 34 A nucleotides (Table 1). As there is more room for A-removal, we decided to make two mutants, the Low and Min variants by removing 42 and 69 A nucleotides, respectively (Table 1). Changes from A to another nucleotide were incorporated when present in at least 5 or 20 of the 675 isolates (0.7–3.0%, respectively).

**Table 1:**
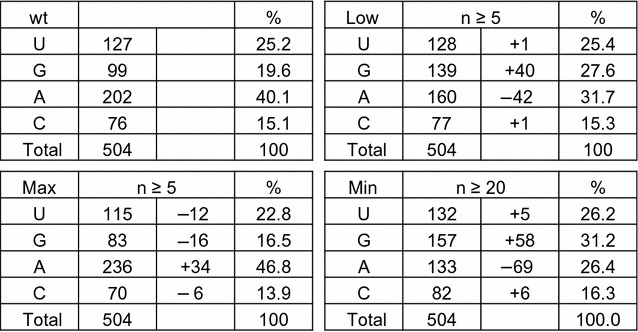
Nucleotide composition of the wt/mutant HIV-1 pol segment

### The nucleotide, dinucleotide and codon characteristics

The basic nucleotide characteristics of these RNA genomes are plotted in Table 1. Of the 498-nucleotide encoded by the Bcl1-Bst1107I fragment, the wt LAI clone contains 200 A nucleotides (40.2%), which was raised to 46.9% for Max and reduced to 31.7 and 26.3% for the Low and Min mutants. A bigger shift is obtained for A-removal than A-addition, which is related to two simple facts. First, the wt starting sequence contains very many As that can be removed. Second, A-addition calls for a specific mutation (N-to-A), whereas A-removal can be towards any other nucleotide (U, C or G). As expected, a concomitant decrease (Max) and increase (Low and Min) was apparent for the non-A nucleotides, although with a non-random distribution. For instance, As were replaced in the Min mutant for G (58×), C (6×) or U (5×). In toto, G-to-A and especially A-to-G changes dominate the mutations chosen, which thus reflects the natural mutational bias of HIV-1 isolates. We believe that this trend is dictated by the characteristics of the codon table. There are eight 4-codon groups where the silent third base can be any nucleotide, but there are also five 2-codon groups where the silent third base change concerns a G/A choice. Another reason for the frequent occurrence of A-to-G over A-to-U/C changes is the observation that transitions are much less frequently observed in HIV-1 evolution than transversions [34, 35]. In fact, we realize that more dramatic silent mutations are possible for the two 6-codon groups, e.g. one could change the CGU codon for Arg silently into AGA and create 2 more As (or remove them in the reversed scenario). We did not implement such multiple changes because they were never observed in the pol gene segment of natural virus isolates (results not shown).

Although we focus in this study on the A-count, other important HIV-1 genome characteristics will also be affected. For instance, studies in diverse viral systems reported an impact on the viral fitness by changes in the dinucleotide count, the codon usage or the codon pair bias [15, 19, 36–38]. As example, we inspected the occurrence of the CG dinucleotide, which is strongly discriminated against in HIV-1 RNA [39]. CG occurs only once in the wt pol gene fragment, whereas the GC dinucleotide occurs 10×, underscoring the strong CG discrimination. The single CG was removed in the Max mutant by a C-to-A mutation, but 4 additional CGs were introduced in the Min mutant, all by A-to-G changes. More changes are likely avoided because other virus isolates also exhibit CG-discrimination. As a control, we also looked at the UA dinucleotide that does not have biased count in HIV-1 RNA [39]. The wt pol gene segment encodes 38 UAs, which goes up to 45 in the Max mutant and down to 20 in the Min mutant. Similar changes are expected for the other genome parameters. Although all changes occur in nature, their clustered appearance is unnatural and may impose problems.

The phylogeny-instructed mutations do obviously have an impact on the codon usage within the mutated pol gene segment that encodes 167 codons. First of all, we like to stress that all changes are based on viral sequences and the codons do not become more human-like or “codon-optimized”. The PIM-introduced synonymous changes by definition affect the synonymous codon bias of the pol segment. This is reflected in a change in the “effective number of codons” [40] or ENC value (results not shown). Closer inspection indicated that the expected nt-changes were present, e.g. towards the use of A-ending codons in the Max mutant. The prevalence of A-ending codons within all 4-codon groups was increased from wt to Max (Ala: 0.60–1.00, Gly: 0.42–0.83, Pro: 0.50–0.86, Thr: 0.45–0.55, Val: 0.64–0.93). The A-count only infrequently reaches saturation (value of 1.00) at these positions, indicating a local restriction in A-saturation in some natural HIV-1 isolates. Local sequence signatures may correlate with distinct cases of A-avoidance.

**Fig. 1:**
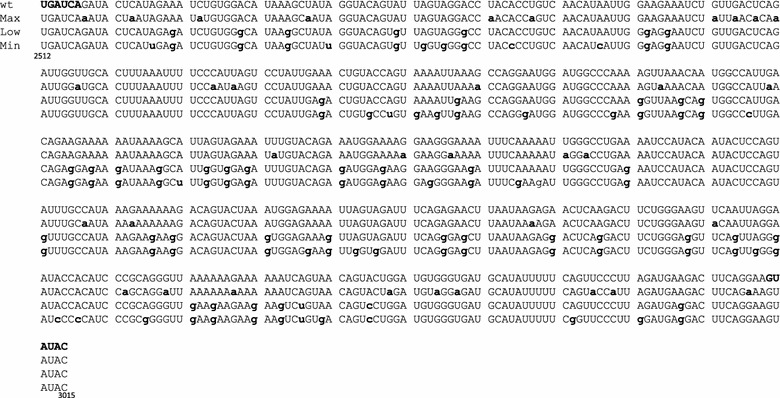
Sequence alignment of the wt LAI and mutant Gag sequences. The 2512–3015 fragment is flanked by restriction enzyme sites (Bcl1-Bst1107I) that are marked in *bold*. Nucleotide positions used for A-insertion (Max) or A-removal (Low and Min) are shown as *small bold letter*

A change away from the A nucleotide was apparent for the Low and especially the Min mutant. Within the 4-codon groups, two distinct patterns were observed. As expected, A is replaced by G in the Val codons (prevalence 0.07–0.46). But all other 4-group codons favored C-ending codons instead. For 3 of these, the presence of C at the second codon position explains the G-avoidance as otherwise a CG dinucleotide would be created (Ala: GCN, Pro: CCN, Thr: ACN). To underscore this scenario, we in fact scored a complete absence of G-ending codons at these three positions in natural virus isolates. The Gly codon group (GGN) favors replacement of the terminal A by C and not by G, which may indicate another local sequence constraint (Fig. 1).

### Nucleotide skew analysis

**Fig. 2:**
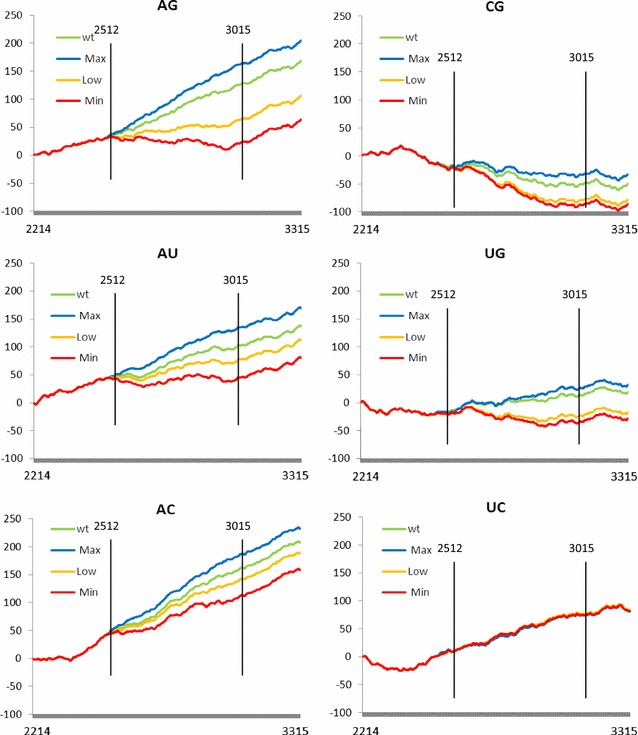
Skew analysis of HIV-1 RNA mutants with an altered A-bias. Skew analysis of the wt and mutant RNA genomes. Skew values (N1 − N2)/(N1 + N2) have been calculated in overlapping windows along the HIV-1 sequence from position 2212 to 3315, which represents the 498-nt Bcl1-Bst1107I fragment with 300-nt wt flanks. Window size was set at 10-nt with a step size of 2-nt, resulting in 550 data points on the *X*-axis. We used the same *Y*-axis for the cumulative skew values to allow a direct comparison of the compositional signatures of the wt and mutant RNA genomes

We next performed a nucleotide skew analysis along the wt and mutant RNA genomes to reveal the nucleotide compositional landscape of this segment (Fig. 2). Skew values were plotted for the 6 comparisons, including 3 with A (A vs G, A vs U, A vs C) along the 498-nt fragment in overlapping windows of 10-nt with a step size of 2-nt. Analysis of the modified fragment included 300-nt 5′ and 3′ flanks, reflecting the wt HIV-1 LAI sequence. As a result, 550 data points were obtained along the analyzed HIV-1 RNA segment. We purposely used the same Y-axis scale for the 6 skew plots to allow a direct comparison of the virus-specific signatures. It should be noted that GA in skew language does not represent a basepair, but a comparison of the number of G-nucleotides with the number of A-nucleotides. The graphs nicely illustrate further enhancement (Max) or attenuation (Low and Min) of the local A-bias. These changes are uniformly distributed over the 498-nt fragment with some minor local fluctuations. The 5′-to-3′ analyses show uniform curve up to position 2512, where the lines start to diverge in the 498-nt fragment, and the lines again move in a parallel manner from position 3015 onwards.

The major effects are observed for the comparisons that include the A-nucleotide (Fig. 2, left hand panels). Among these, the AG skew is most dramatically affected by the A addition/removal, which is explained by the more frequent A–G transition-based mutation in natural HIV-1 isolates that instructed our PIM mutagenesis strategy. For instance, the 34 A-additions of the Max mutant affected G most frequently (16×) over U (12×) and C (6×). These trends are visible in the graphs. The pyrimidine skew (UC) is not at all affected by the A-introduction/removal. Perhaps surprisingly, the CG and UG skews do show a small up-effect for Max and stronger down-effect for Low and Min. These trends also reveal the preferential exchange of A by G (transition) over C/U (transversion). This leads to preferential G depletion in Max, such that the CG and UG skews move up a bit. Bigger effects are observed due to G addition in the Low and Min mutants, which move the CG and UG lines significantly down.

### Viral gene expression

**Fig. 3:**
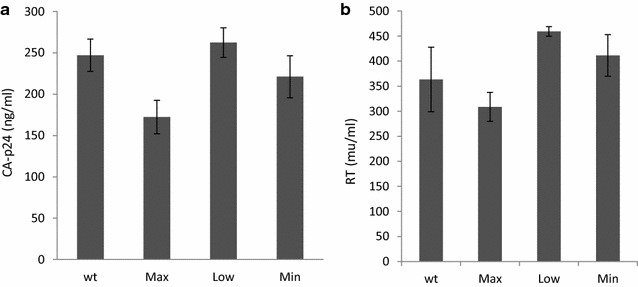
Virus production in DNA-transfected 293T cells. The wt and 3 mutant DNA constructs were transfected in 293T cells and virus production was measured after 2 days by CA-p24 ELISA (**a**) or an RT activity assay (**b**) on the culture supernatant. Average values obtained in three independent transfection experiments are shown with *error bars* indicating standard deviations. Each supernatant sample was analyzed multiple times for CA-p24 (threefold) and RT (twofold). Reduced viral gene expression for Max compared to wt and Low reaches borderline significance (student T test)

We measured virus production in the supernatant of 293T cells transfected with the wt or mutant molecular clones by measuring the CA-p24 level by Elisa. This assay scores the ability of the DNA genomes to be properly expressed, which includes the steps of transcription, RNA splicing needed to synthesize the appropriate level of Tat and Rev protein, and mRNA translation. We scored no profound effect of A-manipulation on these processes, but the Max mutant may be somewhat reduced compared to wt and the other two mutants (Fig. 3a). As an alternative means to measure virus production, we measured the activity of the virion-associated Reverse Transcriptase (RT) enzyme, but no major effect of the A-changes were apparent (Fig. 3b). The minor changes observed likely reflect experimental variation.

### Testing the early phase of virus infectivity

**Fig. 4:**
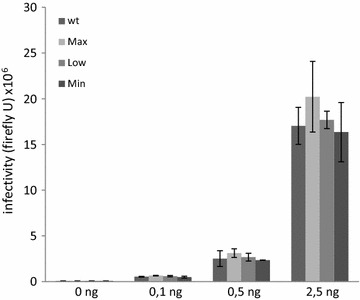
Virus infectivity on TZM-bl cells. We used the 293T-produced virus stocks to measure the infectivity on TZM-bl cells that carry the Tat-responsive luciferase reporter. Average firefly luciferase values obtained in multiple independent experiments are shown with error bars indicating standard deviations (n = 3)

We used to TZM-bl-reporter cells to score the infectivity of the wt and mutant viruses. For this, we used virus that was produced by transfected 293T cells. Equal amounts of virus (based on CA-p24) were used to infect TZM-bl cells and 2 days later we scored the amount of luciferase produced from the Tat-responsive LTR-reporter construct (Fig. 4). No significant differences were scored between the wt and mutant viruses, indicating that the A-manipulation does not affect processes like RNA genome packaging and reverse transcription.

### Spreading infection of A-modified viruses

**Fig. 5:**
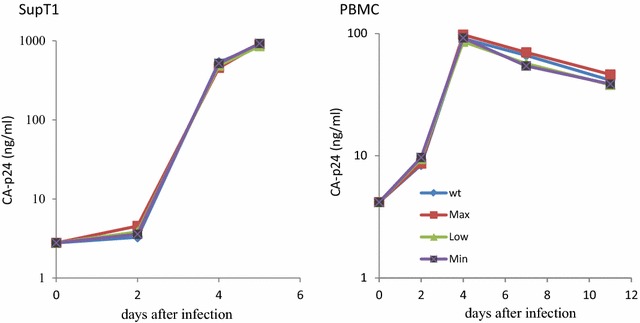
Virus replication on SupT1 T cells and primary T cells. Cells were infected with a standard amount of the wt or mutant viruses (equivalent of 2.5 ng CA-p24 for SupT1 cells and 5 ng CA-p24 for PBMCs). CA-p24 production in the supernatant was monitored longitudinally. Representative replication curves are shown, but similar results were obtained in 3 independent SupT1 and PBMC infections

We next tested the ability of the mutant viruses to replicate in the SupT1 T cell line and primary T cells (Fig. 5). No differences in replication capacity were apparent for these mutants, indicating that silent codon changes designed by PIM allows one to change the viral genome sequence in a neutral manner. We were also interested to verify the genetic stability of the introduced mutations. A priori, one may expect good stability as the mutant viruses replicate as efficiently as the wt LAI virus. We analyzed the sequence of the mutant viruses upon 44–49 days of replication on SupT1 cells. We isolated genomic DNA from these cells and PCR-amplified the integrated proviral sequences. Population-based sequencing indicated that no additional mutations were acquired in the modified pol gene segment for the Low, Min and Max mutants (results not shown), thus testifying to the genetic stability of the PIM-introduced mutations.

### Phylogenetic positioning of the Low, Min and Max mutants

**Fig. 6:**
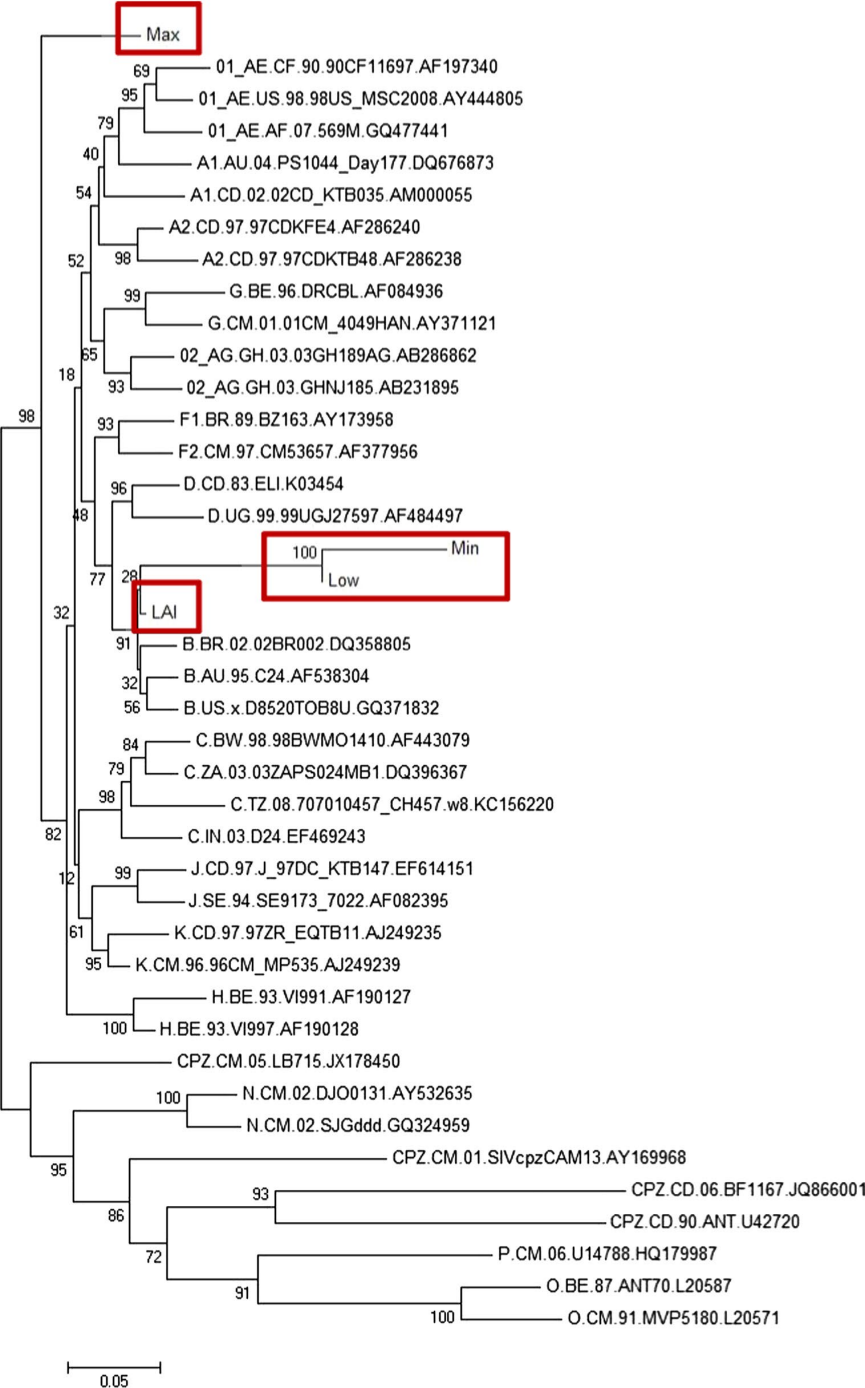
Neighbor-joining tree of the HIV-1 mutants and natural virus isolates based on the 498-nt pol fragment. Reference sequences were selected from several HIV-1 group M subtypes and the distantly related HIV-1N, O and P groups plus several SIV strains from chimpanzee (cpz). Reference sequences are labelled with their Los Alamos Database identifier. The wt LAI isolate and derived Max, Min, and Low mutants are *boxed*. Bootstrap values from 1000 replicates are indicated

Finally, we analyzed the positioning of the A-mutated HIV-1 variants in a phylogenetic tree. To highlight the impact of the changes, we restricted the analysis to the 498-nt pol fragment and made comparisons with virus isolates belonging to different HIV-1 subtypes of the M (major) group. As outgroup we included distant HIV-1 isolates from the N (new), O (outlier) and P groups, but also several SIV strains from chimpanzee (cpz). The pol nucleotide sequences were aligned and MEGA6 (http://www.megasoftware.net) was used to build a phylogenetic tree (Fig. 6). As a control, we also aligned the pol-encoded amino acid sequences, which were identical due to the silent nature of the introduced mutations (not shown).

An initial blast search indicated that the three mutants (Max, Low, Min) stayed within the HIV–SIV cluster of sequence space. Nevertheless, quite remarkable and opposing movements were observed for the three mutants within the HIV–SIV group in the phylogenetic analysis (Fig. 6). Low and Min moved gradually away from the LAI ancestor and Min migrated further as expected because of the more severe A-minimization. The high bootstrap value of 100 at the branching of Low and Min was also anticipated because Min builds further on the Low mutant by removing even more As. The Tamura-Nei distance between LAI and Low (0.099) was nearly doubled for the LAI-Min comparison (0.180). However, both mutants are confidently clustered within subtype B (bootstrap value 91 for the subtype B cluster), with LAI as the closest relative. This result is not unexpected as the PIM approach used subtype B sequence variation to design the changes in the LAI prototype strain.

A contrasting movement within the HIV–SIV tree was apparent for the Max variant, which apparently lost the subtype B-specific signature by taking an “ancestral” position at the base of the complete M group. This result was somewhat unexpected as the PIM approach uses natural subtype B sequences to design the changes. The Tamura-Nei distance for LAI-Max is 0.074, which is in fact less than the distance between LAI and the Low/Min variants. Nevertheless, the A-addition in Max has a larger impact on the positioning in the tree topology. The A-addition in Max does in fact slightly reduce the distance to the SIV cluster (LAI-N is 0.215 and Max-N is 0.176), which is likely due to the fact that A-nucleotides are frequently present at identical positions both in Max and the more distant SIV isolates. We previously reported that a similar A-pressure is apparent in all HIV-1 and SIV strains and subtypes [2].

## Discussion

The presence of naturally occurring sequence variation, especially for viruses like HIV-1 that evolve quickly and for which many isolates have been studied, provides a powerful tool for the description of important sequence and structure motifs. For instance, such a phylogenetic approach has been instrumental in the dissection of critical RNA structures that support HIV-1 replication [41–43]. We now used the sequence information available for natural HIV-1 isolates in the construction of quite massively altered virus genome segments in which the number of A nucleotides was increased from 40.1 to 46.8% or decreased to 26.4%. It is important to stress that although all PIM-mutations were selected based on their occurrence in natural virus isolates, they usually are present as individual mutations, and certainly not clustered as in the PIM-mutated viruses. We report that the designed virus variants are fully replication-competent, demonstrating the power of this PIM as conservative approach. For instance, although the CG dinucleotide is strongly underrepresented in HIV-1 RNA, we could raise the local CG dinucleotide count in the pol segment from 1 to 5 without imposing a significant replication deficit. Other mutagenesis approaches resulted in a significant loss of HIV-1 replication fitness, although the results are difficult to compare because of differences in target gene and extent of the mutated segment.

In this study, we changed a small pol gene segment that comprises 5.5% of the total HIV-1 genome length. Given these promising results, one could consider a more complete rewiring strategy for the 9 kb genome, although it remains a quite elaborate and costly synthetic biology project. Such a holistic approach clearly remains a high-risk procedure as one has to avoid the mutational inactivation of critical replication signals, e.g. the numerous splicing signals (donors, acceptors, enhancers, silencers) or the introduction of toxic signals (e.g. A-addition will frequently introduce stop codons). PIM therefore seems the ideal strategy because all selected mutations have been repeatedly tested and approved in natural isolates. Of course the combination of PIM-assigned changes that are derived from different viruses may still become deleterious, adding to the high-risk. If replication of such a rewired construct is hampered, it would be important to study the interaction of HIV-1 RNA and DNA with components of the innate immune system. One could also use spontaneous virus evolution to repair and thus identify the mutations that most seriously hamper virus replication [44, 45].

When we made such a PIM-rewired HIV-1 genome in silico, it was immediately obvious that certain domains of the HIV-1 genome do not allow many changes, e.g. the Tat–Rev–Env region with gene overlaps (results not shown). One could even envisage an extension of this in silico survey by screening for the absence A nucleotides in this otherwise A-rich genome. These segments may encode yet unknown sequence motifs.

One can think of other applications of the PIM strategy, e.g. the insertion of multiple restriction enzyme sites to facilitate future cloning projects. PIM can be adopted to the study of other viruses, e.g. those with a biased nucleotide composition of their genome. We previously reported that each retrovirus group has its particular nucleotide bias [46], but many other viruses exhibit a biased nucleotide count [47]. For instance, we recently reported generic and group-specific signatures for the human coronaviruses like MERS and SARS [48]. We described a pan-corona bias of A over G, but also a bias of C over U that differs significantly per virus type. Extreme values were apparent with U-counts up to 40.3% and C-counts down to 12.9% for the HKU strain.

The Min/Max results described in this study indicate how far one can go in changing the A-count of the HIV-1 RNA genome without interfering with virus replication, despite the fact that significant repositioning occurred in the phylogeny of these mutants (at least based on the relatively short pol fragment). Nevertheless, such virus variants are fully replication-competent. In other words, we have mapped here the limits of sequence space relative to A-richness in a small pol gene segment that gives rise to a functional HIV-1 genome. We obviously cannot exclude the possibility that the A-count is more critical in other parts of the HIV-1 genome, although A-pressure is intensely present across the genome [2]. This information seems valuable for the design of replication-competent, but attenuated virus variants with numerous mutations that restrict easy reversion to the wt sequence. Such genetically stable hypermutated virus variants could be considered a novel strategy towards a safe live-attenuated vaccine candidate.

We find the Max results perhaps most perplexing for another reason. Using PIM we dramatically altered the phylogenetic position of this LAI variant from within the subtype B cluster to a “primordial” position at the base of the complete M group. However, the Low and Min mutants were in fact equipped with more mutations than Max, but apparently without affecting the core subtype B characteristics. The observation that only the Max mutant lost the subtype B characteristics is intriguing. This differential behavior may hint at what the key determinants are for the subtype-specific signatures. We reason that the key subtype signature is formed by those non-A nucleotides (mostly G) at silent codon positions that fluctuate towards A in other virus isolates as these changes were used to design Max. The driving force behind these decisive changes is likely the striking preponderance of G-to-A changes in HIV-1 evolution [7, 49–51]. As a consequence, other characteristics like genetically stable A and non-A positions and non-silent codon alterations seem less critical for the subtype signature. A related phenomenon has been reported previously for the characteristics that underlie differences among viruses from distinct patient risk groups, in particular homosexual men versus drug users [52]. When silent and non-silent codon positions were analyzed separately, it became apparent that the former deliver the decisive HIV-1 genome characteristics. Although surprising, this finding may suggest that selection at the amino acid level is not the primary driving force for the independent evolutionary behavior of the viral genes.

## Conclusions

We demonstrate that the strongly biased A-count of the HIV-1 RNA genome can be profoundly altered, at least in a small segment of the genome, without imposing a replication penalty. The A-count of a small 498-nt pol fragment was changed from 40.2% (wild-type) to 26.3 or 46.9%. We thus failed to get a handle on a possible replicative function of the A-rich viral genome. At the same time, the full replication potential of the mutant viruses indicates the success of the novel PIM strategy, which proposes silent mutations only when the new nucleotide is repeatedly observed in natural virus isolates. Quite surprisingly, a phylogenetic survey indicated that the mutant with a maximal A-count moved its position to a “primordial” position at the base of all M group HIV-1 subtypes. This result counterintuitively suggests that selection at the amino acid level is not the primary driving force in HIV-1 evolution. Rather, we propose that a key subtype signature is formed by those non-A nucleotides (mostly G) at silent codon positions that regularly oscillate towards A in other virus isolates.

## References

[1] Kypr J, Mrazek J (1987). Unusual codon usage of HIV. Nature.

[2] van der Kuyl AC, Berkhout B (2012). The biased nucleotide composition of the HIV genome: a constant factor in a highly variable virus. Retrovirology.

[3] Berkhout B, van Hemert FJ (1994). The unusual nucleotide content of the HIV RNA genome results in a biased amino acid composition of HIV proteins. Nucleic Acids Res.

[4] Vartanian J-P, Meyerhans A, Sala M, Wain-Hobson S (1994). G - > A hypermutation of the human immunodeficiency virus type 1 genome: evidence for dCTP pool imbalance during reverse transcription. Proc Natl Acad Sci USA.

[5] Martinez MA, Vartanian JP, Wain-Hobson S (1994). Hypermutagenesis of RNA using human immunodeficiency virus type 1 reverse transcriptase and biased dNTP concentrations. Proc Natl Acad Sci USA.

[6] Berkhout B, de Ronde A (2004). APOBEC3G versus reverse transcriptase in the generation of HIV-1 drug-resistance mutations. AIDS.

[7] Pillai SK, Wong JK, Barbour JD (2008). Turning up the volume on mutational pressure: is more of a good thing always better? (A case study of HIV-1 Vif and APOBEC3). Retrovirology.

[8] Deforche K, Camacho R, Laethem KV, Shapiro B, Moreau Y, Rambaut A (2007). Estimating the relative contribution of dNTP pool imbalance and APOBEC3G/3F editing to HIV evolution in vivo. J Comput Biol.

[9] van Hemert FJ, Berkhout B (1995). The tendency of lentiviral open reading frames to become A-rich: constraints imposed by viral genome organization and cellular tRNA availability. J Mol Evol.

[10] Kutluay SB, Zang T, Blanco-Melo D, Powell C, Jannain D, Errando M (2014). Global changes in the RNA binding specificity of HIV-1 gag regulate virion genesis. Cell.

[11] Tanwar HS, Khoo KK, Garvey M, Waddington L, Leis A, Hijnen M (2017). The thermodynamics of Pr55Gag-RNA interaction regulate the assembly of HIV. PLoS Pathog.

[12] Keating CP, Hill MK, Hawkes DJ, Smyth RP, Isel C, Le SY (2009). The A-rich RNA sequences of HIV-1 pol are important for the synthesis of viral cDNA. Nucleic Acids Res.

[13] Deforges J, de Breyne S, Ameur M, Ulryck N, Chamond N, Saadi A (2017). Two ribosome recruitment sites direct multiple translation events within HIV1 Gag open reading frame. Nucleic Acids Res.

[14] Vabret N, Bailly-Bechet M, Najburg V, Muller-Trutwin M, Verrier B, Tangy F (2012). The biased nucleotide composition of HIV-1 triggers type I interferon response and correlates with subtype D increased pathogenicity. PLoS ONE.

[15] Vabret N, Bailly-Bechet M, Lepelley A, Najburg V, Schwartz O, Verrier B (2014). Large-scale nucleotide optimization of simian immunodeficiency virus reduces its capacity to stimulate type I interferon in vitro. J Virol.

[16] Berkhout B, Grigoriev A, Bakker M, Lukashov VV (2002). Codon and amino acid usage in retroviral genomes is consistent with virus-specific nucleotide pressure. AIDS Res Hum Retroviruses.

[17] Kypr J, Mrazek J, Reich J (1989). Nucleotide composition bias and CpG dinucleotide content in the genomes of HIV and HRTLV 1/2. Biochim Biophys Acta.

[18] Li M, Kao E, Gao X, Sandig H, Limmer K, Pavon-Eternod M (2012). Codon-usage-based inhibition of HIV protein synthesis by human schlafen 11. Nature.

[19] Martrus G, Nevot M, Andres C, Clotet B, Martinez MA (2013). Changes in codon-pair bias of human immunodeficiency virus type 1 have profound effects on virus replication in cell culture. Retrovirology.

[20] Hammarskjold ML, Helga-Maria C, Rekosh D, Goff SP (1995). 5′ regions of HIV-1 RNAs are not sufficient for encapsidation: implications for the HIV-1 packaging signal. Virology.

[21] Purcell DFJ, Martin MA (1993). Alternative splicing of human immunodeficiency virus type 1 mRNA modulates viral protein expression, replication, and infectivity. J Virol.

[22] Madsen JM, Stoltzfus CM (2006). A suboptimal 5′ splice site downstream of HIV-1 splice site A1 is required for unspliced viral mRNA accumulation and efficient virus replication. Retrovirology.

[23] Asang C, Erkelenz S, Schaal H (2012). The HIV-1 major splice donor D1 is activated by splicing enhancer elements within the leader region and the p17-inhibitory sequence. Virology.

[24] Klaver B, Berkhout B (1994). Premature strand transfer by the HIV-1 reverse transcriptase during strong-stop DNA synthesis. Nucleic Acids Res.

[25] Klaver B, Berkhout B (1994). Comparison of 5′ and 3′ long terminal repeat promoter function in human immunodeficiency virus. J Virol.

[26] Berkhout B, van Wamel J, Klaver B (1995). Requirements for DNA strand transfer during reverse transcription in mutant HIV-1 virions. J Mol Biol.

[27] Berkhout B, Klaver B, Das AT (1997). Forced evolution of a regulatory RNA helix in the HIV-1 genome. Nucleic Acids Res.

[28] Das AT, Klaver B, Berkhout B (1995). Reduced replication of human immunodeficiency virus type 1 mutants that use reverse transcription primers other than the natural tRNA(3Lys). J Virol.

[29] Das AT, Klaver B, Centlivre M, Harwig A, Ooms M, Page M (2008). Optimization of the doxycycline-dependent simian immunodeficiency virus through in vitro evolution. Retrovirology.

[30] van der Velden GJ, Vink MA, Berkhout B, Das AT (2012). Tat has a dual role in simian immunodeficiency virus transcription. J Gen Virol.

[31] Das AT, Land A, Braakman I, Klaver B, Berkhout B (1999). HIV-1 evolves into a non-syncytium-inducing virus upon prolonged culture in vitro. Virology.

[32] Klaver B, Berkhout B (1994). Evolution of a disrupted TAR RNA hairpin structure in the HIV-1 virus. EMBO J.

[33] Kuiken C, Foley B, Leitner T, Apetrei C, Hahn B, Mizrachi I (2010). HIV sequence compendium 2010.

[34] Keulen W, Boucher C, Berkhout B (1996). Nucleotide substitution patterns can predict the requirements for drug-resistance of HIV-1 proteins. Antiviral Res.

[35] Keulen W, Nijhuis M, Schuurman R, Berkhout B, Boucher CAB (1997). Reverse transcriptase fidelity and HIV-1 variation. Science.

[36] Atkinson NJ, Witteveldt J, Evans DJ, Simmonds P (2014). The influence of CpG and UpA dinucleotide frequencies on RNA virus replication and characterization of the innate cellular pathways underlying virus attenuation and enhanced replication. Nucleic Acids Res.

[37] Greenbaum BD, Levine AJ, Bhanot G, Rabadan R (2008). Patterns of evolution and host gene mimicry in influenza and other RNA viruses. PLoS Pathog.

[38] Vabret N, Bhardwaj N, Greenbaum BD (2017). Sequence-specific sensing of nucleic acids. Trends Immunol.

[39] Berkhout B (1996). Structure and function of the human immunodeficiency virus leader RNA. Prog Nucleic Acid Res Mol Biol.

[40] Wright F (1990). The ‘effective number of codons’ used in a gene. Gene.

[41] Berkhout B (1992). Structural features in TAR RNA of human and simian immunodeficiency viruses: a phylogenetic analysis. Nucleic Acids Res.

[42] Paillart JC, Marquet R, Skripkin E, Ehresmann B, Ehresmann C (1994). Mutational analysis of the bipartite dimer linkage structure of human immunodeficiency virus type 1 genomic RNA. J Biol Chem.

[43] van Bel N, Das AT, Berkhout B (2014). In vivo SELEX of single-stranded domains in the HIV-1 leader RNA. J Virol.

[44] Das AT, Berkhout B (2010). HIV-1 evolution: frustrating therapies, but disclosing molecular mechanisms. Philos Trans R Soc Lond B Biol Sci.

[45] Berkhout B, Das AT (2009). Virus evolution as a tool to study HIV-1 biology. Methods Mol Biol.

[46] van Hemert F, van der Kuyl AC, Berkhout B (2014). On the nucleotide composition and structure of retroviral RNA genomes. Virus Res.

[47] van Hemert F, van der Kuyl AC, Berkhout B (2016). Impact of the biased nucleotide composition of viral RNA genomes on RNA structure and codon usage. J Gen Virol.

[48] Berkhout B, van Hemert F (2015). On the biased nucleotide composition of the human coronavirus RNA genome. Virus Res.

[49] Keulen W, Back NKT, van Wijk A, Boucher CAB, Berkhout B (1997). Initial appearance of the 184Ile variant in lamivudine-treated patients is caused by the mutational bias of the human immunodeficiency virus type 1 reverse transcriptase. J Virol.

[50] Armitage AE, Deforche K, Chang CH, Wee E, Kramer B, Welch JJ (2012). APOBEC3G-induced hypermutation of human immunodeficiency virus type-1 is typically a discrete “all or nothing” phenomenon. PLoS Genet.

[51] Wood N, Bhattacharya T, Keele BF, Giorgi E, Liu M, Gaschen B (2009). HIV evolution in early infection: selection pressures, patterns of insertion and deletion, and the impact of APOBEC. PLoS Pathog.

[52] Lukashov VV, Jurriaans S, Bakker M, Berkhout B (2013). Transmission of risk-group specific HIV-1 strains among Dutch drug users for more than 20 years and their replacement by nonspecific strains after switching to low-harm drug practices. J Acquir Immune Defic Syndr.

